# Peripherally induced Foxp3^+^ regulatory T cells mediates the immunomodulatory effect of intravenous immunoglobulin in an experimental model of allergic airway disease

**DOI:** 10.1186/1710-1492-10-S1-A50

**Published:** 2014-03-03

**Authors:** Amir H Massoud, Gabriel Kaufman, Madelaine Taylor, Marianne Beland, Ciriaco A Piccirillo, Walid M Mourad, Bruce D Mazer

**Affiliations:** 1 McGill University, Dept. of Experimental Medicine, Montreal, QC, Canada, H2X 2P2; 2 McGill University Dept. of Microbiology and Immunology, Montreal, QC, Canada, H3G 1A4; 3 Universite de Montral, Dept. Microbiologie et Immunologie Montreal, QC, Canada, H2X 3J4

## Background

IVIg is a polyclonal IgG preparation with potent immune-modulating properties. We demonstrated that IVIg protects against airway hyperreactivity (AHR) and airway inflammation in mouse models of allergic airway disease, accompanied by peripheral induction of Foxp3^+^regulatory T-cells (iT_reg_). The requirement of IVIg-induced iT_reg_ and their antigen-specificity in attenuation of AHR and airway inflammation remains unknown.

## Methods

We utilized DEREG mice, carrying a transgenic diphtheria toxin receptor under the control of the Foxp3 promoter, allowing for selective depletion of Foxp3^+^T_reg_ by the application of diphtheria toxin (DT). Mice were sensitized and challenged with ovalbumin (OVA) and treated with IVIg. AHR was measured using a FlexiVent small animal ventilator. Total and antigen-specific IgE, as well as pro-inflammatory cytokines levels were determined in serum and alveolar lavage, using ELISA.

## Results

In the absence of Treg, due to multiple DT doses before and after the treatment, IVIg was not able to attenuate AHR, diminish IgE levels and Th-2 type cytokine production, nor alleviate airway inflammation. However, mice in which the pre-established T_reg_ cells (nT_reg_) were depleted before but not following IVIg treatment demonstrated an induction of Foxp3^+^T_reg_ to IVIg therapy and did not develop AHR and airway inflammation to allergen-challenge. Adoptive transfer of enriched IVIg-induced iT_reg_ from OVA-IVIg treated mice failed to transfer protection to mice exposed to ragweed, but was protective in OVA-sensitized and challenged mice.

## Conclusions

T_reg_ can be induced from effector CD4^+^T-cells in the absence of nT_reg_. IVIg-induced antigen specific T_reg_ are capable of suppressing all aspects of antigen-driven airway inflammation in an antigen-specific manner.

